# B cells expressing IL-10 mRNA modulate memory T cells after DNA-Hsp65 immunization

**DOI:** 10.1590/1414-431X20154409

**Published:** 2015-09-18

**Authors:** I. C. Fontoura, A.P.F. Trombone, L. P. Almeida, J. C. C. Lorenzi, R. A. M. Rossetti, T. Malardo, E. Padilha, W. Schluchting, R. L. L. Silva, A. F. Gembre, J. E. C. Fiuza, C. L. Silva, A. Panunto-Castelo, A. A. M. Coelho-Castelo

**Affiliations:** 1 Faculdade de Medicina de Ribeirão Preto, Universidade de São Paulo, Ribeirão Preto, SP, Brasil; 2 Universidade Sagrado Coração, Bauru, SP, Brasil; 3 Instituto de Ciências Biomédicas, Universidade de São Paulo, São Paulo, SP, Brasil; 4 Departamento de Educação em Saúde, Universidade Federal de Sergipe, Lagarto, SE, Brasil; 5 Faculdade de Filosofia, Ciências e Letras de Ribeirão Preto, Universidade de São Paulo, Ribeirão Preto, SP, Brasil; 6 Universidade Paranaense, Cascavel, PR, Brasil

**Keywords:** DNA-Hsp65 vaccine, Memory T cells, B cells

## Abstract

In DNA vaccines, the gene of interest is cloned into a bacterial plasmid that is engineered to induce protein production for long periods in eukaryotic cells. Previous research has shown that the intramuscular immunization of BALB/c mice with a naked plasmid DNA fragment encoding the *Mycobacterium leprae* 65-kDa heat-shock protein (pcDNA3-Hsp65) induces protection against *M. tuberculosis* challenge. A key stage in the protective immune response after immunization is the generation of memory T cells. Previously, we have shown that B cells capture plasmid DNA-Hsp65 and thereby modulate the formation of CD8^+^ memory T cells after *M. tuberculosis* challenge in mice. Therefore, clarifying how B cells act as part of the protective immune response after DNA immunization is important for the development of more-effective vaccines. The aim of this study was to investigate the mechanisms by which B cells modulate memory T cells after DNA-Hsp65 immunization. C57BL/6 and BKO mice were injected three times, at 15-day intervals, with 100 µg naked pcDNA-Hsp65 per mouse. Thirty days after immunization, the percentages of effector memory T (TEM) cells (CD4^+^ and CD8^+^/CD44^high^/CD62L^low^) and memory CD8^+^ T cells (CD8^+^/CD44^high^/CD62L^low^/CD127^+^) were measured with flow cytometry. Interferon γ, interleukin 12 (IL-12), and IL-10 mRNAs were also quantified in whole spleen cells and purified B cells (CD43^−^) with real-time qPCR. Our data suggest that a B-cell subpopulation expressing IL-10 downregulated proinflammatory cytokine expression in the spleen, increasing the survival of CD4^+^ TEM cells and CD8^+^ TEM/CD127^+^ cells.

## Introduction

DNA vaccines consist of a gene of interest cloned into a bacterial plasmid, which is then further engineered to express the protein for long periods in eukaryotic cells (1). In this way, nucleic acids can be used to induce a specific immune response against a pathogen, offering a wide range of new options in vaccinology (2). In previous studies, the intramuscular immunization of BALB/c mice with a naked DNA fragment encoding *Mycobacterium leprae* 65-kDa heat-shock protein (pcDNA3-Hsp65) imparted protection against *M. tuberculosis*challenge (3,4).

The DNA vaccine construct ensures protein production within those cells that capture the plasmid DNA. Thereafter, the antigens in the cytoplasm become accessible to the proteasomal pathway and can be presented via MHC I to activate CD8^+^T cells. Cytotoxic T lymphocytes are a key part of the protective immune response to intracellular pathogens (5). A proportion of the endogenously produced antigen can also be secreted or released upon cell death, and captured by another antigen-presenting cell (APC). Professional APCs can also present antigens derived from the Hsp65 in an MHC II context, activating specific CD4^+^ T cells, a mechanism called “cross-presentation” (5). Moreover, CpG motifs in the plasmid sequence induce an innate immune response, activated by toll-like receptor 9 (TLR9), which in turn elicits specific T-cell functions (6).

Specific memory lymphocytes are generated during the immune response. Central memory T cells recirculate through the secondary lymphoid organs, as the host immune surveillance mechanism, whereas effector memory T cells (TEM) rapidly differentiate into effector cells upon exposure to the antigen for a second time, and thus play an important role in the protective immune response. Therefore, the main goal in vaccine design is the generation of long-lived memory T cells. Some factors that contribute to memory T-cell maintenance are the cytokines interleukin 12 (IL-12) and IL-15, and also IL-7, which plays an essential role in the maintenance of CD8^+^ memory cells. These cytokines induce the proliferation and antiapoptotic mechanisms of memory T cells, allowing them to survive for extended periods (7).

Although the main function attributed to B cells is antibody production, they are also professional APCs, and can capture, process, and present soluble antigens, leading to the activation of CD4^+^ T lymphocytes and consequently enhancing the CD8^+^ cytotoxic response (8). A central role of B cells in maintaining CD4^+^ and CD8^+^ T cells for long periods in mice infected with *Plasmodium chabaudi*, *Listeria monocytogenes*, or *Lymphocytic choriomeningitis virus*(LCMV) has previously been demonstrated (9,10). However, their role in memory induction during DNA immunization is not well understood.

We have previously demonstrated that B cells captured the plasmid pcDNA3-Hsp65 (11), presented the expressed protein, and modulated the memory CD8^+^ T cells formation after mice were challenged with *M. tuberculosis* (12). However, clarifying the specific mechanisms by which B cells induce a protective immune response after DNA immunization is an important step in the development of more-effective DNA vaccines.

Here, we investigated the mechanisms by which B cells modulate memory T cells in the pcDNA3-Hsp65 vaccinated mouse model. Our results showed that a B-cell subpopulation expressing IL-10 mRNA downregulated the expression of proinflammatory cytokines, thus increasing the percentages of CD4^+^ and CD8^+^memory T cells in the spleen after DNA immunization.

## Material and Methods

### Mice

Male 6-8-week-old C57BL/6 wild-type (WT) and B-cell-deficient (BKO; μ chain^−/-^) mice were obtained from Jackson Laboratories (USA) and maintained under specific-pathogen-free conditions in the animal house of the Departamento de Imunologia, Faculdade de Medicina de Ribeirão Preto, Universidade de São Paulo. The mice had access to water and sterile food *ad libitum*, and were maintained under light cycles of 12 h. The protocol used for animal experimentation was approved by the Institutional Committee for Animal Use (CETEA-FMRP-USP; process #040/2006).

### Immunization

The quadriceps muscle of each mouse was injected three times, at 15-day intervals, with 100 µg naked pcDNA3-Hsp65 (DNA-Hsp65) or pcDNA3 (empty vector) in 25% PBS-sucrose, in a total volume of 100 µL. DNA-Hsp65 and the vector were prepared as described previously (4). The mice were euthanized 30 days after the last dose. Other mice were injected with 100 µL saline as a control. All experiments were performed with four animals per treatment group.

### Phenotyping memory T cells

The spleens were aseptically removed from the immunized mice and the spleen cells were restimulated with 20 µg recombinant Hsp65 in RPMI 1640 medium (Invitrogen, USA) containing 10% heat-inactivated fetal bovine serum (FBS) (Gibco BRL, USA), 100 U/mL penicillin, 100 mg/mL streptomycin, and 10 mg/mL gentamicin (Sigma Aldrich, USA) for 24 h. The cells were stained with anti-CD4, anti-CD62L, anti-CD127, anti-CD8 or anti-CD4 antibodies (BD Bioscience, USA). The labeled cells were analyzed with flow cytometry (FACSCanto™, BD Bioscience).

### Real-time RT-PCR

After stimulation, the spleen cells were collected with 1 mL of TRIzol Reagent (Invitrogen) and their total RNA was extracted according to the manufacturer’s protocol.

On day 30 after immunization, the B cells were separated from the spleens using negative selection (>90% CD19^+^ cells) with an anti-CD43 antibody linked to magnetic beads (MACS MicroBeads System, Miltenyi Biotec, Germany). The B cells were collected with 1 mL of TRIzol Reagent (Invitrogen) and their total RNA was extracted according to the manufacturer’s protocol.

The total RNA was treated with amplification-grade DNase I (Invitrogen). Complementary DNA (cDNA) was reverse transcribed from the mRNA with SuperScript II (Gibco BRL), according to the manufacturer’s instructions. The real-time qPCR reactions were performed with 200 ng cDNA, 0.1 µg/µL of each primer (sense and antisense), and Platinum SYBR Green qPCR SuperMix-UDG (Invitrogen), according to the manufacturer’s instructions, on a Rotor-Gene 6000 (Corbett Life Science, Australia). Relative expression was calculated as 2^−ΔΔCt^ (13). The annealing temperature used was 58°C for all genes. The following primers sequences were used: beta-actin: forward 5′-AGCTGCGTTTTACACCCTTT-3′, reverse 3′AAGCCATGCCAATGTTGTCT-5′; IL-12 p40: forward 5′AGCACCAGCTTCTTCATCAGG-3′, reverse 3′-GCGCTGGATTCGAACAAAG-5′; interferon γ (IFN-γ): forward 5′-GATATCTGGAGGAACTGGCAA-3′, reverse 3′-GCTCTGCAGGATTTTCATGTC-5′; IL-10: forward 5′-TGGACAACATACTGCTAACCG-3′, reverse 3′-GGA TCATTTCCGATAAGGCT-5′.

### Statistical analyses

Statistical analyses were performed with one-way analysis of variance (ANOVA), followed by Tukey’s test. Differences with P values less than 0.05 were considered to be statistically significant.

## Results

### Percentage of memory T cells increased in mouse spleens after DNA immunization

**Figure 1:**
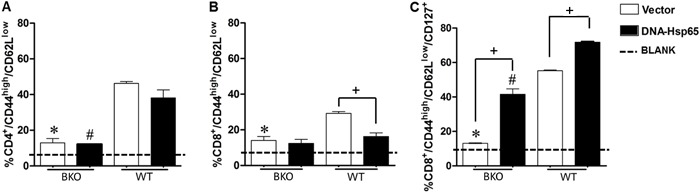
Percentage of CD4^+^ and CD8^+^ effector memory T cells (TEM) in the spleens of wild-type (WT) and B-cell knockout (BKO) mice 30 days after immunization. C57BL/6 WT and BKO mice were immunized three times with 100 µg naked pcDNA3 encoding *Mycobacterium leprae* 65-kDa heat-shock protein (DNA-Hsp65 group). Some animals were immunized three times with 100 µg empty pcDNA3 or 100 µL of saline (0.9%) as a control (Vector and BLANK groups). Mice were immunized intramuscularly at 15-day intervals. Thirty days after the last immunization, the spleens were harvested and the phenotypes of *A*, CD4^+^TEM, *B*, CD8^+^ TEM, and *C*, CD8^+^ TEM expressing CD127 were analyzed with flow cytometry. *,#P<0.05: BKO immunized with DNA-Hsp65 or Vector *vs* WT immunized with DNA-Hsp65 or Vector; ^+^P<0.05: BKO or WT immunized with DNA-Hsp65 *vs* BKO or WT immunized with Vector (ANOVA, followed by Tukey’s test).

To verify that the absence of B cells plays a negative role in memory generation after DNA-Hsp65 immunization, the percentages of CD4^+^ and CD8^+^ TEM cells were analyzed in the mouse spleens 30 days after the last vaccination. Our data showed a higher percentage of CD4^+^/CD44^high^/CD62L^low^ cells in the WT mice than in the BKO mice after immunization (Figure 1A). However, the percentages of CD4^+^ TEM cells did not differ significantly between the groups immunized with pcDNA3-Hsp65 or empty vector in both the WT and BKO immunized mice (Figure 1A). The WT group immunized with empty vector showed a higher percentage of CD8^+^/CD44^high^/CD62L^low^ cells than the BKO mice immunized with empty vector. However, the percentage of CD8^+^/CD44^high^/CD62L^low^ cells in the WT and BKO mice immunized with pcDNA3-Hsp65 did not differ significantly (Figure 1B). Because no significant differences were found between the CD8^+^ TEM subpopulations in the spleens of the WT and BKO mice immunized with pcDNA3-Hsp65, we evaluated the CD8^+^ TEM cells expressing CD127^+^, a specific marker of memory CD8^+^ T cells (14). We found that when mice were immunized with either DNA-Hsp65 or empty vector, the WT mouse spleens had higher percentages of CD8^+^ memory cells than the spleens of the BKO mice. In contrast to CD8^+^ TEM, pcDNA3-Hsp65 immunization induced a higher percentage of CD8^+^ TEM/CD127^+^ cells than the empty vector in both the WT and BKO mice (Figure 1C). Taken together, these data suggest that the presence of B cells contributed to the induction of memory CD4^+^ and CD8^+^ T cells in the spleen after immunization with plasmid DNA.

### WT mice displayed reduced proinflammatory cytokine mRNAs in the spleen

**Figure 2:**
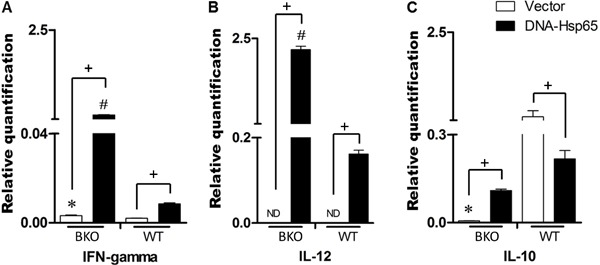
Relative expression of cytokine mRNAs in the spleens of wild-type (WT) and B-cell knockout (BKO) mice 30 days after immunization. C57BL/6 WT and BKO mice were injected intramuscularly on three occasions, at 15-day intervals, with 100 µg pcDNA3 encoding *Mycobacterium leprae* 65-kDa heat-shock protein (DNA-Hsp65 group) or with 100 µg empty pcDNA3 as a control (Vector group). Thirty days after the last immunization, the splenic gene profiles of *A*, IFN-γ, *B*, IL-12, and *C*, IL-10 were evaluated with real-time qPCR. ND: not detected. *^,#^P<0.05: BKO immunized with DNA-Hsp65 or Vector *vs* WT immunized with DNA-Hsp65 or Vector; ^+^P<0.05: BKO or WT immunized with DNA-Hsp65 *vs* BKO or WT immunized with Vector (ANOVA, followed by Tukey’s test).

When the transcriptional profiles of the proinflammatory cytokines in the mouse spleens were evaluated 30 days after immunization, we found that DNA-Hsp65 immunization increased the mRNA levels of IFN-γ and IL-12 compared with empty-vector immunization in both the WT and BKO mice (Figure 2A and B, respectively). It is noteworthy that transcripts of IFN-γ and IL-12 were virtually undetectable in the cells of mice immunized with the empty vector. The WT mice showed lower mRNA expression of IFN-γ and IL-12 after DNA-Hsp65 immunization than the BKO mice. In contrast, no significant difference in IL-10 mRNA expression was observed between the WT and BKO groups when the mice were immunized with DNA-Hsp65. IL-10 mRNA expression was only elevated in the WT group immunized with the empty pcDNA3 vector (Figure 2C). These data indicate a role for B cells in the regulation of proinflammatory cytokine production in the mouse spleen.

### DNA-Hsp65 immunization induced IL-10 mRNA expression by B cells

**Figure 3:**
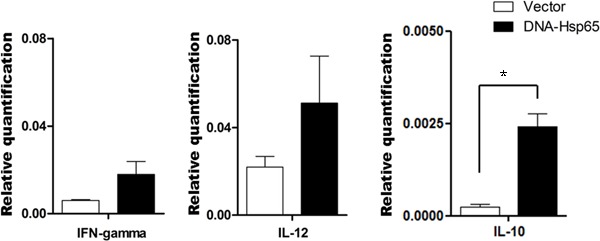
Relative expression of cytokine mRNAs in purified B cells from wild-type (WT) mouse spleens 30 days after immunization. C57BL/6 WT mice were injected intramuscularly on three occasions, at 15-day intervals, with 100 µg pcDNA3 encoding *Mycobacterium leprae*65-kDa heat-shock protein (DNA-Hsp65 group) or with 100 µg empty pcDNA3 as a control (Vector group). Thirty days after the last immunization, the B cells were purified from the spleens of WT mice with magnetic beads, using an anti-CD43 monoclonal antibody (negative selection). Gene profiles of *A*, IFN-γ, *B*, IL-12, and *C*, IL-10 in B cells (CD43^−^) were evaluated with real-time qPCR. *P<0.05 WT immunized with DNA-Hsp65 *vs* WT immunized with Vector (ANOVA, followed by Tukey’s test).

To clarify the possible mechanisms by which B cells modulate the formation of memory T cells and regulate proinflammatory cytokine expression, the mRNA expression of IFN-γ, IL-12, and IL-10 was measured in B cells purified from mouse spleen cells 30 days after immunization. The splenic B cells from mice immunized with DNA-Hsp65 or empty vector showed similar levels of IFN-γ and IL-12 mRNA expression (Figure 3A and B, respectively). However, B cells from the DNA-Hsp65-immunized mice displayed higher levels of IL-10 mRNA than the B cells from the empty-vector-immunized mice. This suggests that DNA-Hsp65 immunization activates a subpopulation of B cells that produces IL-10.

## Discussion

Our results suggest that the presence of B cells is necessary to support the formation of memory after DNA immunization. Memory T cells develop after the evolution of the adaptive immune response. This protective response begins after the recognition of the antigen presented by professional APCs to naïve T lymphocytes, which triggers their proliferation and differentiation into effector T cells. After antigen clearance, the immune response is downregulated and most activated lymphocytes undergo apoptosis. The pool of remaining lymphocytes then differentiates into long-lived memory T cells (15). A previous study showed that as well as presenting antigens, B cells also costimulate T cells through their interaction with CD40 and CD40L on the T-cell surface, enhancing T-cell activation (16). Additional costimulation by their engagement with CD28 induces greater T-cell survival in the effector phase of the immune response, by promoting an increase in antiapoptotic molecules in the activated T cells. This event allows a larger number of the available cells to differentiate into memory cells (17). Our results corroborate these previous reports, because we observed a higher percentage of CD4^+^ and CD8^+^ T cells in the WT mouse spleen after DNA immunization. These data indicate that B lymphocytes have an important function in promoting the costimulation of T cells, resulting in a higher percentage of memory cells 30 days after immunization. Although no significant difference in the percentage of CD8^+^ TEM cells was observed between the WT and BKO spleens, there was a two-fold reduction in the percentage of CD8^+^TEM/CD127^+^ cells in the BKO mice compared with the WT mice over the same period after DNA immunization. CD127 corresponds to the alpha chain in the receptor of IL-7, an important cytokine in the maintenance of memory T cells in the peripheral immune system (18). The CD8^+^ T cells that maintain their CD127 expression after the expansion of the immune response are destined to become memory T cells (7). Therefore, the evaluation of CD127 expression on CD8^+^ TEM cells allows us to evaluate the specific, long-lived CD8^+^ T cells generated after DNA vaccination.

Our data provide further support for the key role of B cells in improving memory formation by promoting T-cell costimulation, and also demonstrate that DNA immunization induces a subtype of IL-10-producing B cells that reduces the amount of proinflammatory cytokine mRNAs in the spleen. Although IL-10 is considered a regulatory cytokine, we have shown that this cytokine is required for the formation of memory cells after LCMV infection. In the presence of this cytokine, the resolution of infection occurred later, causing antigen persistence, and thus stimulating the adaptive immune response for a long period. This consequently activated a larger number of memory T cells (19). The induction of sufficient immunological memory depends on the magnitude of the immune response generated against the antigen. Therefore, in an exacerbated inflammatory environment, T cells are highly activated and consequently undergo apoptosis, reducing the memory pool. Under these circumstances, the activation of an IL-10-producing B-cell subpopulation after DNA immunization is important for the modulation of the immune response induced by the DNA vaccine.

Recently, many studies have demonstrated the regulatory role of IL-10-producing B cells (reviewed by Balkwill et al., 20). Although this role has been described in autoimmune diseases, this B-cell subpopulation also inhibited the activation of myeloid cells *in vitro*, suggesting that these cells have a role in maintaining the homeostasis of the immune system. Although it is difficult to determine the exact phenotype of regulatory B cells, many efforts have been made to investigate the characteristics of these cells. Several studies have demonstrated that their regulatory function could be a transient phenotype of those B cells designated to be antibody-secreting cells, which is induced when they are stimulated with TLR agonists in the absence of B-cell antigen receptor stimulation. Under these conditions, B cells produce IL-10, leading to the regulation of the immune response (20).

In conclusion, our data suggest the participation of IL-10-producing B cells in the immune response by modulating the induction of the immune response after the presentation of DNA-Hsp65 by impairing the exacerbation of the inflammatory response. This may improve the longevity of the antigen derived from Hsp65 in the spleen, thus improving the activation of a protective immune response.
